# Distinct Dynamics of Endocytic Clathrin-Coated Pits and Coated Plaques

**DOI:** 10.1371/journal.pbio.1000191

**Published:** 2009-09-08

**Authors:** Saveez Saffarian, Emanuele Cocucci, Tomas Kirchhausen

**Affiliations:** Department of Cell Biology, Harvard Medical School, Children's Hospital and Immune Disease Institute, Boston, Massachusetts, United States of America; Princeton University, United States of America

## Abstract

Clathrin is the scaffold of a conserved molecular machinery that has evolved to capture membrane patches, which then pinch off to become traffic carriers. These carriers are the principal vehicles of receptor-mediated endocytosis and are the major route of traffic from plasma membrane to endosomes. We report here the use of in vivo imaging data, obtained from spinning disk confocal and total internal reflection fluorescence microscopy, to distinguish between two modes of endocytic clathrin coat formation, which we designate as “coated pits” and “coated plaques.” Coated pits are small, rapidly forming structures that deform the underlying membrane by progressive recruitment of clathrin, adaptors, and other regulatory proteins. They ultimately close off and bud inward to form coated vesicles. Coated plaques are longer-lived structures with larger and less sharply curved coats; their clathrin lattices do not close off, but instead move inward from the cell surface shortly before membrane fission. Local remodeling of actin filaments is essential for the formation, inward movement, and dissolution of plaques, but it is not required for normal formation and budding of coated pits in the cells we have studied. We conclude that there are at least two distinct modes of clathrin coat formation at the plasma membrane—classical coated pits and coated plaques—and that these two assemblies interact quite differently with other intracellular structures.

## Abstract 2

Here we classify endocytic structures at the adherent (bottom) surface of many cells in culture into shorter-lived coated pits and longer-lived coated plaques which internalize by different mechanisms.

## Author Summary

Here, we identify and characterize two distinct modes of clathrin-mediated uptake at the plasma membrane. The “canonical” coated pit is where assembly of a curved clathrin lattice, linked to deformation of the underlying membrane, gives rise to coated vesicles. Clathrin coated “plaques” are extended clathrin lattices of low curvature (enriched in hexagonal arrays) that have been observed by electron microscopy at the bottom of cells, but their relationship to the canonical, curved pit assembly has been obscure. Recognition of the difference between two distinguishable classes of events detected by fluorescence microscopy has resolved a number of conflicts and misconceptions in the literature. In particular, a large fraction of the clathrin endocytic processes studied at the adherent surface of HeLa, Swiss 3T3, and astrocyte cells are the long-lived coated plaques, not canonical coated pits, whereas most, if not all, of the clathrin endocytic processes at the free surface of these cells are coated pits. Conflation of data from the two distinct processes has previously led to misleading mechanistic conclusions, which are now resolved.

## Introduction

Clathrin-mediated endocytosis is a mechanism for selective retrieval and internalization of membrane lipids and membrane-bound proteins. Clathrin-coated pits capture their molecular cargo as they invaginate from the cell surface and bud inward to form coated vesicles, in a process that involves a complex sequence of interactions among structural and regulatory proteins and lipids [1]–[3].

The assembly of clathrin-coated pits can be followed in vivo by contemporary live-cell imaging methods, including laser scanning confocal fluorescence microscopy, spinning disk confocal microscopy, and a combination of wide-field (WF) and total internal reflection (TIR) fluorescence microscopy [4]–[16]. These studies have yielded an array of data and models, in part depending on the cell type or imaging method uses, and a range of different views of how coats form, of potential roles for actin and the cytoskeleton, and of possible mechanistic similarities between clathrin-based structures in yeast and mammalian cells (reviewed in [1],[17],[18]).

A combination of high- and moderate-resolution molecular structures from X-ray crystallography and electron cryomicroscopy (cryoEM) and dynamic data from live-cell fluorescence imaging of BSC1 cells [5],[14],[19]–[22] has led to the following picture for coated pit formation. At widely distributed locations on the plasma membrane, sequential recruitment of clathrin and its adaptors progressively bends the underlying bilayer as a clathrin lattice assembles; the adaptors selectively capture membrane-bound proteins destined for endocytosis; membrane pinching separates the fully formed coated vesicle from its parent membrane; and an ATP-dependent chaperone uncoats the released vesicle. Important features of this process in BSC1 cells are that most of the endocytic structures are smaller than the diffraction limit of the optical microscope (∼250 nm) and that the coated pits form progressively, over a time between nucleation and pinching of 35–65 s [5].

In other types of cells—HeLa and COS cells in particular—there are, in addition to the rapidly forming coated pits with characteristics similar to those in BSC1 cells, larger and more stable clathrin structures. The latter are present only on the “bottom” (adherent, coverslip proximal) surface of the cell, and we initially thought that they might represent clathrin reservoirs [5]. Relatively flat, extended clathrin arrays, referred to as “clathrin plaques” [23], have been observed by conventional electron microscopy on the bottom surface of HeLa cells and osteoclasts. These plaques probably correspond to the extended hexagonal lattices of clathrin seen in these and other cell types (e.g., Swiss 3T3 cells) when imaged by rapid-freeze, deep-etch electron microscopy [24]–[27]. We believe that the plaques also correspond to the relatively long-lived clathrin structures (lifetimes >3 min) at the substrate-facing surfaces of primary adipose cells imaged in real time by TIR [28], of Swiss 3T3 cells imaged in real time by TIR and WF fluorescence illumination [9],[10], and of HeLa and COS cells as described above [5]. Many of these long-lived clathrin structures display an abrupt inward movement at the end of their lifetime—a process associated with membrane internalization and transferrin uptake [9],[10]. Because no firm distinction has previously been made between the dynamics of clathrin-coated pits and plaques, the observations just summarized have spawned a model in which preformed clathrin lattices on the plasma membrane undergo an extensive structural reorganization, leading to coat curvature, membrane invagination, and vesicle budding [18],[29].

Different results and interpretations on the potential role of actin in clathrin-based endocytosis have accompanied these fundamentally different models for the mechanics of coat assembly. A connection between actin and clathrin-driven membrane uptake was first described in yeast [30],[31]. Acute depolymerization of actin by latrunculin A inhibits clathrin-based endocytosis in some metazoan cells, but not in others [32]. Latrunculin treatment has no detectable effect on the dynamics of endocytic coated pits in BSC1 cells [33], but it strongly inhibits endocytosis in Swiss 3T3 cells, with a marked reduction in the number of clathrin-based endocytic events [15]. Similarly, perturbation of the actin cytoskeleton by genetic means, such as depletion of Hip1R [34], perturbations in Hip1R association with cortactin [8], and depletion of N-WASP [35], has variable effects that depend on cell type. Some clathrin endocytic structures recruit actin or cortactin at the time they bud and disappear, but others, in the same cells, do not [9],[10],[15],[36]–[38].

In an effort to differentiate among distinct modes of clathrin-mediated endocytosis, we have measured the properties of clathrin assemblies on both free (top) and adherent (bottom) lower surfaces of several mammalian cell types. Recently introduced methods in live-cell imaging enable us to make distinctions more readily than has previously been possible. We find that a coated pit grows continuously as an invaginating shell, which pinches off immediately upon completion, as in the usual pictures. The entire process typically takes 30–60 s. A coated plaque grows initially at about the same rate as a coated pit, but without displacement from the cell surface. Its growth reaches a fluctuating plateau, generally two to three times that of a typical pit, remaining in place for up to several minutes before moving uniformly inward a few seconds before membrane pinching. The actin cytoskeleton is not required for normal formation and budding of the rapidly growing pits, but it is essential for the formation, inward movement, and dissolution of plaques. We have thus distinguished two modes of clathrin-coat formation at the plasma membrane, with quite different mechanisms for coat internalization. That is, clathrin is a scaffold for at least two distinct, membrane-associated processes. The distinction allows us to resolve apparent contradictions in the previous literature of clathrin-mediated endocytosis.

## Results

### Comparison of Clathrin Dynamics on the Upper and Lower Surfaces of Swiss 3T3 Cells

**Figure 1:**
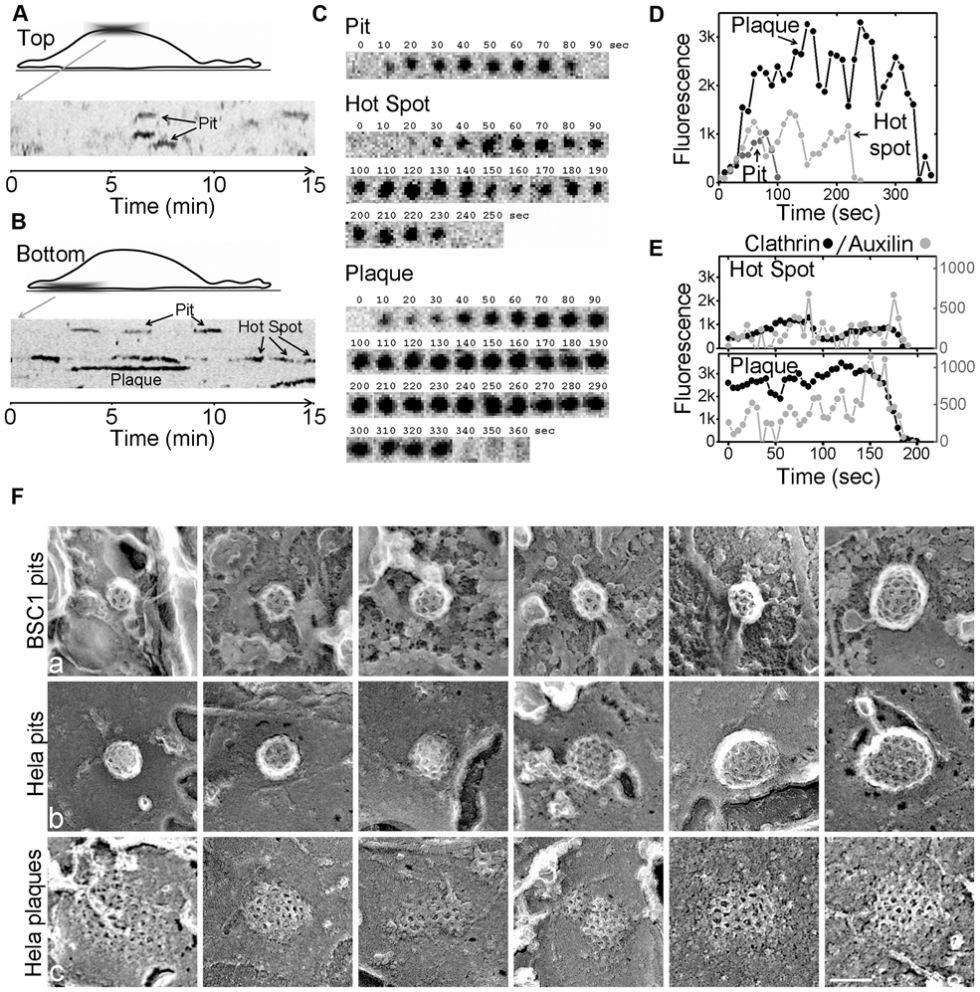
Formation of clathrin-coated structures on the plasma membrane of cells. (A–E) Time-series images of clathrin assemblies at the top or bottom of Swiss-3T3 cells acquired with a spinning disk confocal microscope (Videos S1 and S2). The fluorescent assemblies were tagged with clathrin LCa-DsRed stably expressed in Swiss-3T3 [9] in the absence (A–D) or presence (E) of transiently expressed auxilin1-EGFP. (A) Representative kymograph obtained from a time series recorded every 5 s (100-ms exposure) for 15 min from the free (top) surface of the cell, showing that the majority of the structures are relatively short-lived. (B) Representative kymograph obtained from a time series recorded every 5 s (100-ms exposure) for 15 min from the adherent (bottom) surface of the cell. All structures are dynamic, but many have longer average lifetimes than those in (A). (C) Selected snapshots from the time series shown in (B) to illustrate examples of clathrin structures displaying the dynamic behavior characteristic of canonical coated pits, of a group of canonical coated pits forming consecutively at a single location (hot spot), and of a plaque. The peak fluorescence intensity of pits assembling in isolation is similar to those forming as part of a hot spot; the overall intensity of the plaque is significantly higher. The acquisition time of each snapshot (in seconds) is indicated. (D) Fluorescence intensity profiles of the clathrin assemblies shown in (C). (E) Fluorescence intensity profiles of clathrin coats tagged with LCa-DsRed and Auxilin1-EGFP to illustrate the dynamics of canonical pits forming in a hot spot and of plaques. A burst of auxilin recruitment coincides with the uncoating step of a canonical pit [22],[43], or with each of the uncoating steps observed within a hot spot; in contrast, variable amounts of auxilin are recruited during the entire life of the plaque, ending with a larger burst. (F) Electron microscopy of clathrin-coated structures on the adherent surface of unroofed BSC1 and HeLa cells. Representative electron micrographs illustrate in (a) the exclusive presence of clathrin-coated pits with various degree of invagination in the plasma membrane of unroofed BSC1 cells; 79 pits were captured in 49 pictures (156 µm^2^). Representative electron micrographs illustrate in (b and c) the coexistence of clathrin-coated pits and clathrin flat arrays in the plasma membrane of unroofed HeLa cells. 69 pits and 48 sheets were captured in 59 pictures (188 µm^2^); Bar indicates 100 nm.

We compared the dynamics of clathrin-containing assemblies on the free surface of Swiss 3T3 cells with their dynamics at the surface in contact with the coverslip (Figure 1 and Videos S1 and S2). For these experiments, we used a previously described cell line in which clathrin is labeled by stable expression of LCa-dsRed [9], and we recorded images using spinning disk confocal imaging. We restricted our analysis to objects that were relatively stationary in *x* and *y*, as most of the more motile ones correspond to endosomes [5],[7]. By using exposure times of 100 ms per frame and imaging every 2 to 10 s, we could record for periods of up to 60 min without obvious signs of phototoxicity. All experiments reported here were carried out under conditions in which internalization of transferrin, used as a probe of clathrin-dependent uptake, was not affected by expression of the fluorescent chimeric proteins.

**Figure 2:**
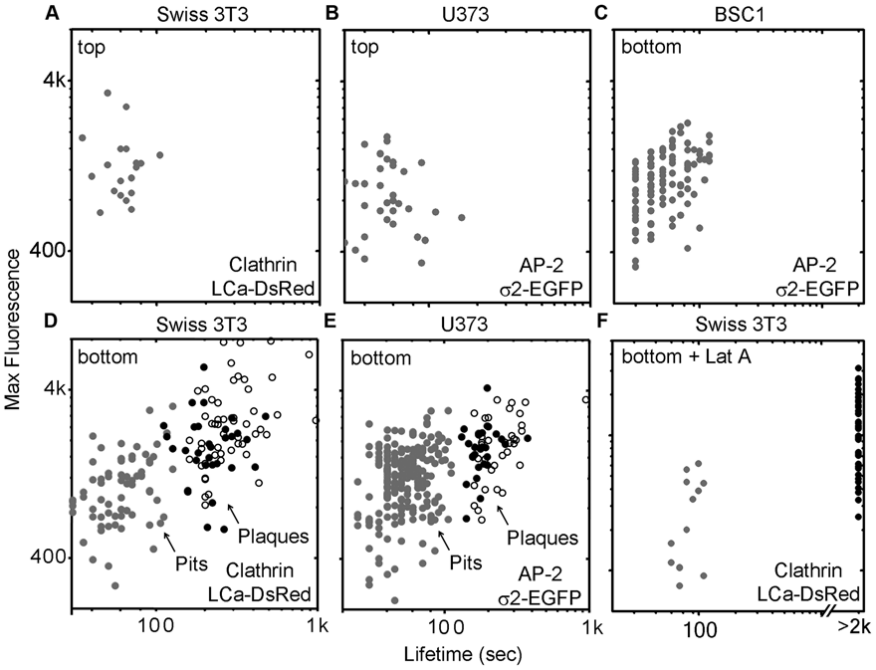
Sizes and lifetimes of clathrin-coated structures forming at the cell surfaces. In these double logarithmic plots, each dot corresponds to the duration (lifetime) and the corresponding size (maximum fluorescence intensity prior to dissolution) of a clathrin or AP-2 fluorescent spot recorded at the top or bottom plasma membrane of adherent cells. The data derive from time series (100-ms exposures) of at least 15-min duration recorded every 5 s (full circles) or 60-min duration recorded every 10 s (empty circles) using a spinning disk confocal fluorescence microscope. The dots are color coded to distinguish clathrin coats displaying dynamics characteristics of canonical pits (gray) from those behaving as plaques (black). The time series were acquired from Swiss 3T3 stably expressing clathrin LCa-DsRed, or from U373 astrocytes and BSC1 cells stably expressing AP-2 σ2-EGFP. Similar results were obtained from data acquired in two additional Swiss, two additional astrocytes, and two additional BSC1 cells (Figure S4). (A and D) The average lifetime of canonical pits tagged with LCa-DsRed is 62±16 s (*n* = 19) on the top of the Swiss 3T3 cells and 64±24 s (*n* = 71) on the bottom; the average lifetime of plaques (bottom only) is longer and more variable (271±141 s; *n* = 87). The corresponding maximum fluorescence intensities are 1,357±698, 1,130±564, and 2,542±1,784, respectively. The differences in average lifetime or of maximum fluorescence intensity between pits and plaques in Swiss 3T3 cells are statistically significant (*p*<0.0001) (B, C, and E) The average lifetimes of canonical pits tagged with σ2-EGFP of AP-2 is 60±26 s (*n* = 34) and 56±19 s (*n* = 221) on the top and on the bottom of U373 astrocytes, and 56±26 s (*n* = 105) on the bottom of BSC1 cells. The lifetime of the AP-2 containing plaques on the bottom of astrocytes is 219±112 s (*n* = 67). The corresponding maximum fluorescence intensities are 932±431, 1,109±428, and 2,542±1,784, respectively. The differences in average lifetime or of maximum fluorescence intensity between pits and plaques in astrocytes are statistically significant (*p*<0.0001). (F) Effect on clathrin-coat dynamics of actin cytoskeleton depolymerization by 15-min treatment of Swiss 3T3 cells expressing LCa-DsRed with 5 µM latrunculin A. The average lifetime (84±18 sec; *n* = 12) and maximum fluorescence intensity (1,178±775; *n* = 41) of the canonical coated pits remains constant, while the number of newly formed pits decreases. In contrast, the plaques became stationary, with average lifetimes in excess of 20 min (*p*<0.0001), and there is a decrease in the number of newly formed plaques.

Nearly all the fluorescent spots on the free surface of the Swiss 3T3 cells belonged to a single class of diffraction-limited objects, as expected for coated pits or vesicles <200 nm in diameter (Figure 1A). These spots displayed the growth and abrupt disappearance of fluorescence, characteristic of assembling clathrin-coated pits [5],[14]. In general, newly forming pits appeared at positions uncorrelated with previous events—that is, “hotspots” were rare or absent (Figure 1C and 1D). The mean lifetime of these structures was 62±16 s, with an average maximum intensity of approximately 800 fluorescence units, recorded just prior to their disappearance (Figure 2A). These values are similar to those determined for conventional clathrin pits that form on the top or bottom surfaces of astrocytes, BSC1, COS, or HeLa cells, stably or transiently expressing light chain a (LCa) fused to mRFP, YFP, EGFP, Tomato, Cherry, or DsRed [5],[14],[22],[33]. Clathrin-coated pits, visualized in human U373 astrocytes by stable expression of σ2-EGFP (the AP-2 small chain), also have the same characteristics (Figure 2B); the σ2-EGFP colocalizes completely with clathrin tagged with Tomato-LCa (unpublished data). An advantage of marking endocytic coated pits with AP-2 is that this adaptor complex is not directed to any intracellular membranes. Its use in our previous work with BSC1 cells therefore eliminated confusion from recorded events due to endosomes or other intracellular structures [5],[14],[22],[33],[39].

Clathrin-containing structures at the adherent surface of the Swiss 3T3 cells have a more complex pattern of formation and dissolution. They can be divided into three categories, each with distinct dynamic properties (Figure 1B–1D). Those in the first category have the same time course as described above for conventional coated pits and vesicles (Figure 1C, pit, and 1D, dark gray tracing; Figure 2D, gray dots). The intensity profiles of structures in the second category are similar to those in the first, but they form sequentially at the same positions (hotspots) (Figure 1D). A new spot starts to appear before full disappearance of the previous one. The number of sequential events is variable, and the positions of their sites of initiation cannot be resolved (i.e., are less than 500 nm apart) (Figure 1B–1E). This hotspot behavior has been described by others for structures on the adherent surface of Swiss 3T3 [10],[15] and COS cells [5],[6].

Clathrin-containing structures in the third category, which we term *clathrin-coated plaques* (borrowing a phrase from Maupin and Pollard [23]), have substantially longer lifetimes (typically 2–16 min) (Figures 1B–1E, 2D, and 2E, black dots and circles). Quantitative determination of their lifetimes was possible, because of the longer imaging periods we used for this work. The clathrin fluorescence associated with coated plaques is, on average, stronger than the fluorescence of conventional endocytic coated pits, whether the pits arise at random positions or at hotspots on the coverslip proximal surface (Figure 1D). The intensity of coated plaques often undergoes prominent fluctuations (see Figure 1D and 1E), in agreement with similar observations from large clathrin ensembles located at membrane in contact with the coverslip [9],[10],[28]. The rate of clathrin accumulation at the beginning of assembly is roughly the same for pits and plaques, and the uncoating phase of 6–10 s is likewise similar in the two cases, but the rate at which clathrin accumulates during fluctuations at later times in the life of a plaque is often greater. We also detected clathrin-coated plaques, visualized with tagged AP-2, on the adherent surfaces of U373 astrocytes stably expressing σ2-EGFP (Figure 2E, black dots; see the comparison in the dynamics of coat formation on the adherent surface of BSC1 and U3T73 cells in Video S4), and long-lived clathrin structures are present on the adherent surfaces of COS and HeLa cells (unpublished data). The absence of detectable AP-2 hot spots or plaques on the free surface of astrocytes (Figure 2B) agrees with earlier observations showing that similar structures are absent from the nonadherent surfaces of BSC1, HeLa cells, or astrocytes [5],[22],[33]. We estimate that the relative amounts of clathrin engaged in coated plaques and coated pits, respectively, at the attached surface of a cell after overnight plating are 70% and 30% for Swiss 3T3, 65% and 35% for U373, and 80% and 20% for HeLa cells. As described below, the endocytic traffic capacity of plaques is relatively small, because they are only present on the adherent surface and because the frequency of plaque-mediated endocytic events (pinching off) is significantly lower than that of coated-pit mediated.

To correlate the coated pits and coated plaques, as defined above by their properties when observed by live-cell imaging, with morphologies seen by electron microscopy, we visualized the inward-facing surface of adherent plasma membranes from “unroofed” BSC1 and HeLa cells by the well-established methods of rapid freezing, freeze etching, and rotary shadowing [40]. Adherent surfaces of BSC1 cells contain coated pits but essentially no plaques, whereas the corresponding surfaces of HeLa cells contain both. We detect only curved clathrin coats of various sizes and degrees of invagination in BSC1 cells that by live-cell imaging have only coated pits (Figure 1Fa: 70 pits in a total surface area of 156 µm^2^; 49 electron microscopy images). In contrast, we find both invaginated pits (Figure 1Fb: 67 pits in a total surface area of 188 µm^2^; 59 electron microscopy images) and relatively shallow sheets (Figure 1Fc: 48 sheets in the same area) on the adherent surface of HeLa cells. (Because of limitations in the way cells are treated to reveal the inward-facing surface of the adherent membrane, these micrographs correspond to views from the thinnest, most peripheral regions of the attached cell surface, where the relative number of plaques seen by optical microscopy is substantially lower than in the center; the count of 48 sheets and 67 pits, therefore, considerably underestimates the plaque:pit ratio across the full adherent surface.) We conclude that clathrin-coated pits and coated plaques as defined by live-cell imaging criteria correspond respectively to the canonical coated pits and extended sheets observed by classical electron microscopy.

To probe whether AP-2 is essential for plaque formation, we depleted AP-2 in HeLa cells stably expressing σ2-EGFP (to monitor AP-2) and transiently expressing Tomato-LCa (to monitor clathrin). We used RNA interference (RNAi) specific for μ2 [41] for a period of approximately 5 d, with a double transfection protocol (Materials and Methods) that gave full ablation of fluorescent transferrin uptake in more than 90% of the cells and a 10-fold reduction in the number of clathrin structures, as described before [41]. Under these conditions, clathrin and AP-2 were completely absent from the top surface of the cells (unpublished data). Immobile, diffraction-limited, long-lived (>500 s) clathrin structures were present on the adherent surface, however (Video S5). These spots contained a very small amount of AP-2, with a fluorescent signal barely above background—less than 2% of the signal from plaques in HeLa cells not depleted of AP-2. As there were no conventional coated pits on the top or bottom surface of the cells, we interpret the immobile structures on the bottom surface of AP-2–depleted cells as nascent plaques that were unable to grow, because they had exhausted the extremely limited amount of residual AP-2 present in those cells. We also detected motile clathrin-coated structures in the cytosol and perinuclear regions, with the usual characteristics of endosome- or TGN-associated coats containing AP-1, AP-3, GGAs, or Hrs. We conclude that AP-2 is essential for the formation of conventional coated pits and probably also for the initiation and growth of coated plaques.

### Recruitment of Auxilin

Auxilins 1 and 2 are J-domain cofactors for Hsc70, the uncoating ATPase [42]. Auxilins appear in coated vesicles in a characteristic burst that lasts several seconds, just at the onset of disassembly of the clathrin/AP-2 coat [22],[43]. We find similar bursts of transiently expressed auxilin-1-EGFP in coated vesicles of the LCa-DsRed–expressing Swiss 3T3 cells (Figure 1E), and we can record auxilin bursts at the onset of each of the serial dissolutions of the clathrin signals observed during the lifetime of a hotspot (Figure 1E). Small, variable levels of auxilin are also present in association with clathrin during the lifetime of conventional coated pits and hotspots. These common features of isolated pits and hotspots support the interpretation that the latter correspond to sequential assembly and independent budding of conventional coated pits and vesicles, at positions that all lie within the resolution limit of the microscope. In contrast, coated plaques recruit substantially greater quantities of auxilin during their lifetime and at the onset of their dissolution than do the conventional structures (Figure 1E). We could not detect any direct correlation between variations in clathrin and auxilin fluorescence during the lifetime of the plaques (Figure 1E and unpublished data). We conclude that sequential formation of conventional pits and vesicles and their detachment from a plaque is not an adequate explanation for the fluctuations in clathrin and AP-2 seen during the lifetime of the plaques.

### Role of Actin

Coated pits and plaques differ particularly strikingly in the relationship between their properties and the dynamic state of actin. Treatment of Swiss 3T3 cells with latrunculin A, which depolymerizes the actin cytoskeleton, prevents both formation of new plaques and dissolution of old ones but has no effect on the dynamics of assembly and dissolution of conventional clathrin-coated pits (Figure 2F, Figure S1, and Video S6). These observations confirm our earlier work on clathrin dynamics in BSC1 cells (which lack plaques) [33]. We showed that formation of conventional clathrin-coated pits is not sensitive to loss of actin dynamics after treatment with either latrunculin A or cytochalasin D. Other groups have reported that treatment with latrunculin A of Swiss 3T3 cells (which contain both pits and plaques) prevents coat dissolution at the adhered surface [15]—presumably because the observations were weighted heavily toward the coated plaques.

There are two known connections between actin and clathrin assemblies in cells. One is huntingtin-interacting protein 1-related (Hip1R), which has binding sites for F-actin, cortactin, and clathrin light chains [44]–[47]. Interference with the interaction between Hip1R and clathrin light chains by overexpression of a light-chain mutant unable to associate with Hip1R retains the cation-independent mannose-6-phosphate receptor (CI-MPR) in the TGN but has no detectable effect on transferrin or EGF uptake [47]. The other connection between clathrin coats and actin dynamics involves cortactin, which activates the actin nucleation factor Arp2/3 and leads to stimulation of branched actin filament assembly [48]–[50]. It is believed that dynamin recruits cortactin through interaction of the proline-rich C-terminus of dynamin with the SH3-domain of cortactin [51].

**Figure 3:**
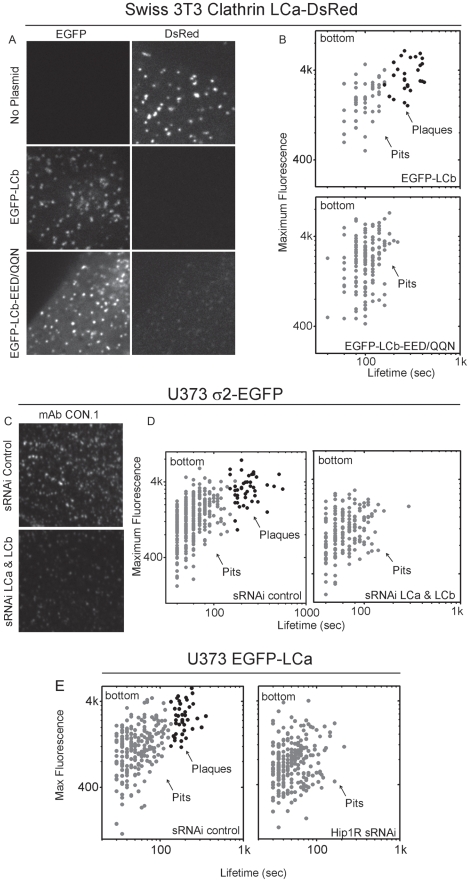
Disruption of plaque formation by interference with the function of clathrin light chains. Loss of plaques upon expression of EFGP-LCb-EED/QQN (A and B) or due to depletion of clathrin light chains or Hip1R by RNAi (C, D, and E). (A) Effective replacement of LCa-DsRed with EGFP-LCb or EFGP-LCb-EED/QQN. Swiss 3T3 cells stably expressing LCa-DsRed were transfected with a modified form of clathrin EGFP-LCb, in which the conserved three critical residues EED required for the interaction of the light chains with Hip1R were mutated to QQN. As control, cells were transfected with no plasmid or with wild-type EGFP-LCb. The fluorescence images show the characteristic punctate pattern of clathrin light chains elicited by the LCa constructs and, due to replacement, the corresponding loss of LCb fluorescence. The estimated replacement level is 85% (*n* = 170). (B) Expression of EGFP-LCb-EED/QQN prevents formation of plaques, but not of pits. The panels are representative semi-logarithmic plots of maximum fluorescence versus time for pits and plaques from two Swiss 3T3 cells expressing similar amounts of EGFP-LCb or EGFP-LCb-EED/QQN. The average lifetime of canonical pits on the bottom of the Swiss 3T3 cells expressing EGFP-LCb is 106±31 s (*n* = 45) and 107±31 s (*n* = 71) in cells expressing EFGP-LCb-EED/QQN. The maximum fluorescence intensities of these pits are 2,131±968 and 2,526±1,392, respectively. The average lifetime and maximum fluorescence intensity of plaques on the bottom of the Swiss 3T3 cells expressing EGFP-LCb are 291±76 s and 3,701±1,451 (*n* = 27). The differences in average lifetime or in maximum fluorescence intensity between pits and plaques in cells expressing EGFP-LCb is statistically significant (*p*<0.0001). There is no statistically significant difference in average lifetime or in maximum fluorescence intensity between pits of cells expressing EGFP-LCb or EFGP-LCb-EED/QQN. (C and D) Loss of plaques upon depletion of clathrin light chains. Both clathrin light chains of U373 astrocytes were depleted by transfection with Oligofectamin using a pool of sRNAi specific for the light chains. As control, cells were transfected with Oligofectamin using a scrambled sRNAi sequence (Materials and Methods). (C) The extent of light-chain depletion (∼85%) was estimated using mAb CON.1 immunofluorescence labeling; the mAb recognizes both light chains. (D) The panels are representative semi-logarithm plots of maximum fluorescence versus time for pits and plaques from two different cells containing normal or depleted amounts of clathrin light chains. The average lifetime and maximum fluorescence intensity of the pits in the control cell were 63±23 and 1,562±888 (*n* = 304), respectively. The average lifetime and maximum fluorescence intensity of the pits in the cell depleted of clathrin light chains were 68±31 and 1,259±589 (*n* = 168), respectively; the differences are not statistically significant. The average lifetime and maximum fluorescence intensity of the plaques in the control cell were 243±91 and 3,396±1,388 (*n* = 43), respectively; the differences in average lifetime and in maximum fluorescence intensity between pits and plaques in the control cells are statistically significant (*p*<0.0001). The plaques are absent in the cell depleted of clathrin light chains. (E) Loss of plaques upon depletion of Hip1R. Hip1R of U373 astrocytes transiently expressing EGFP-LCa were depleted by transfection with Oligofectamin using a pool of sRNAi specific for Hip1R. As control, cells were transfected with Oligofectamin using a scrambled sRNAi sequence (Materials and Methods). The panels are representative semi-logarithmic plots of maximum fluorescence versus time for pits and plaques from two different cells containing normal or depleted amounts of Hip1R. The average lifetime and maximum fluorescence intensity of the pits in the control cell were 58±26 and 1,334±761 (*n* = 209), respectively. The average lifetime and maximum fluorescence intensity of the pits in the cell depleted of Hip1R were 56±27 and 866±570 (*n* = 237), respectively; the differences are not statistically significant. The average lifetime and maximum fluorescence intensity of the plaques in the control cell were 194±47 and 2,840±1,133 (*n* = 36), respectively; the differences in average lifetime and in maximum fluorescence intensity between pits and plaques in the control cells are statistically significant (*p*<0.0001). Plaques are absent in the cell depleted of Hip1R.

**Figure 4:**
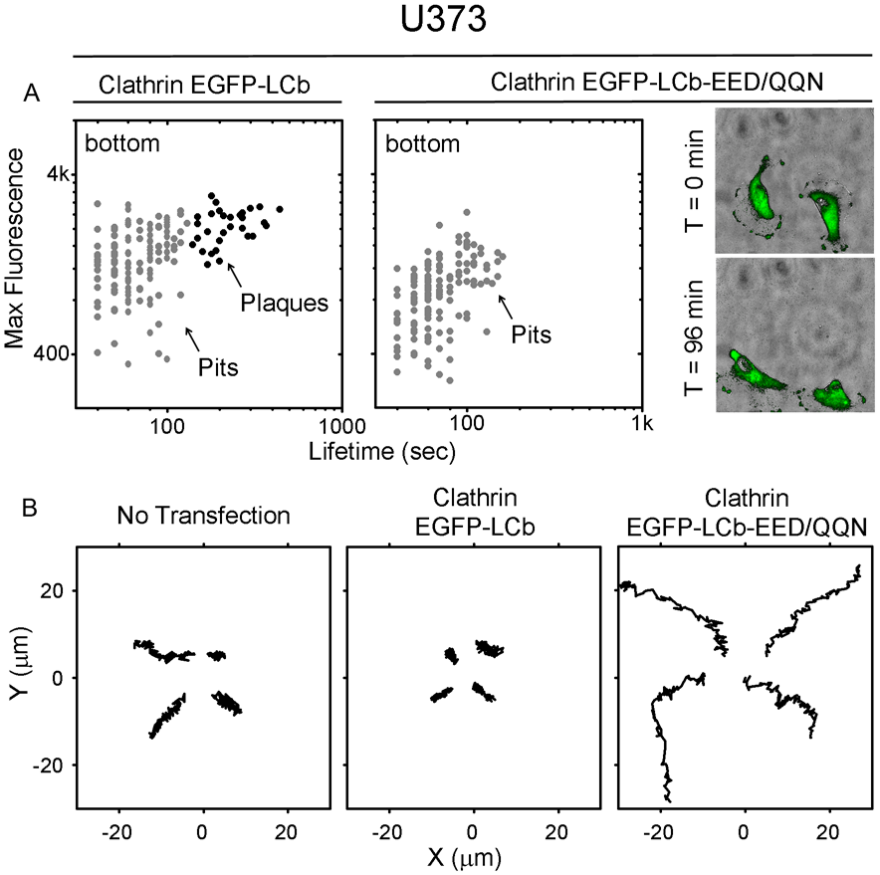
Increase of cell motility correlates with loss of plaques. (A) Expression in U373 astrocytes of EGFP-LCb-EED/QQN prevents plaque formation. U373 astrocytes were transfected with EGFP-LCb-EED/QQN or with EGFP-LCb as control, and their clathrin structures imaged 48 h after transfection. The semilogarithmic plots represent maximum fluorescence versus time of pits and plaques from two cells expressing similar amounts of EGFP-LCb or EGFP-LCb-EED/QQN. The average lifetimes are 69±23 s (*n* = 129) for canonical pits tagged with EGFP-LCb is and 232±78 s (n = 30) for plaques; the corresponding fluorescence intensity maxima are 1,420±536 and 2,087±455, respectively. The difference in maximum fluorescence intensity is statistically significant (*p*<0.0001). The right panels are snapshots from two different time points of a video acquired from cells expressing EGFP-LCb-EED/QQN using the WF fluorescence combined with bright-field phase illumination; the images show the elongated shape of the cells and the change in their position. Control cells (unpublished data) are more symmetric and remain stationary. (B) Increased motility of cells correlates with absence of plaques. Each panel shows four motility tracks, each from a different cell that either expresses its endogenous clathrin light chains (not transfected), or where they have been replaced, by overexpression, with EGFP-LCb or EGFP-LCb-EED/QQN.

To disrupt the interaction between clathrin and Hip1R, we used the dominant-negative clathrin LCb-EED/QQN fused to EGFP [47], a mutant protein that binds normally to clathrin heavy chain but is deficient in its interaction with Hip1R [44],[46] We found that transient overexpression of EGFP-LCb-EED/QQN in Swiss 3T3 cells stably expressing LCb-DsRed or in U373 astrocytes resulted in complete elimination of plaques but that it had no discernable effects on the formation and properties of normal pits (Figures 3B and 4A). In agreement with earlier observations obtained with HeLa cells [47], overexpression of EGFP-LCb-EED/QQN had no discernable effects on the clathrin-dependent receptor-mediated uptake of fluorescent transferrin in astrocytes (Figure S5), a result that is consistent with the small relative contribution of plaques to endocytic traffic (see below). As expected, the mutant light chain colocalized with and replaced wild-type LCa-DsRed (Figure 3A), with an efficiency of approximately 85% as determined by the extent in the decrease of the fluorescence signal of LCa-DsRed at any clathrin spot. Control experiments performed by expression of wild-type EGFP-LCb showed no effects on the formation of clathrin plaques or pits (Figures 3B and 4A) even though the replacement level was similar to that achieved with EGFP-LCb EED/QQN (Figure 3A).

The role of Hip1R in plaque formation was confirmed by the loss of plaques, but not of pits in cells depleted of Hip1R by RNAi (Figure 3E). Under these conditions, the average lifetime and maximum fluorescence intensity of the remaining pits at the bottom surface do not change.

Depletion of clathrin LCa and light chain b (LCb) is another way to disrupt the linkage between clathrin and Hip1R because it also leads to accumulation of CI-MPR in the TGN without affecting uptake of transferrin or EGF [47],[52]. To test whether loss of light chains also results in absence of plaques, we depleted LCa and LCb in U373 cells stably expressing σ2-EGFP (Figure 3C antibody staining) by using specific small RNA interference (sRNAi) probes to both light chains [47],[52]. We found a complete absence of coated plaques, whereas formation of coated pits remained normal (Figure 3D).

In the course of the light chain replacement experiments performed by expression of EGFP-LCb EED/QQN, we saw a clear association between absence of plaques and a marked increase in cell motility (Figure 4A and 4B). This increase was not due merely to expression of exogenous light chains, as expression of wild-type EGFP-LCb had no detectable effect (Figure 4B).

**Figure 5:**
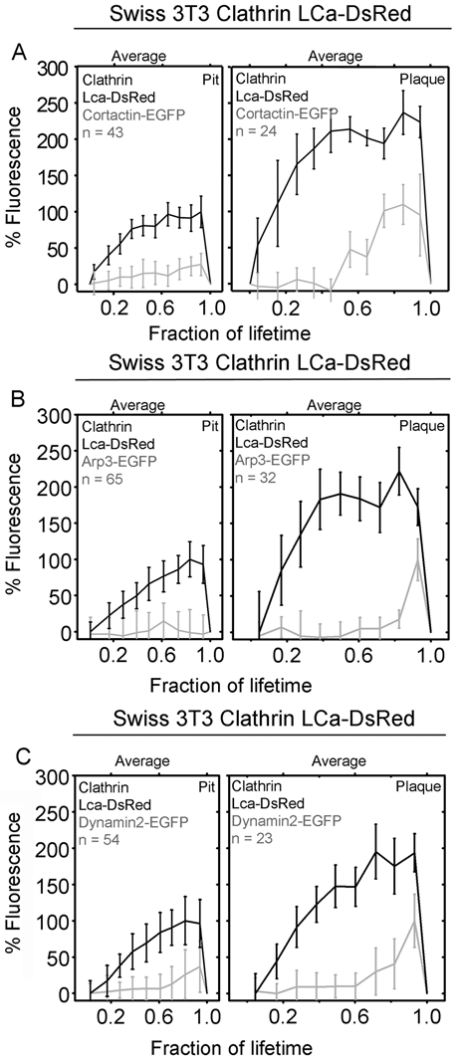
Recruitment of cortactin, Arp2/3, and dynamin into clathrin-coated pits and plaques. Spinning disk fluorescence intensity profiles obtained from time-series images of clathrin assemblies forming at the adherent (bottom) surface of Swiss 3T3 fibroblasts stably expressing Clathrin LCa-DsRed and transiently expressing (A) cortactin-EGFP, (B) Arp3-EGFP, and (C) dynamin2-EGFP. Fluorescence images for each set of these proteins were simultaneously acquired with a beam splitter using 200-ms exposures obtained every 5 s. The points represent averages of complete datasets, normalized to the maximum intensity and to the lifetime of the object. Each point represents average plus or minus standard deviation. *N* is the number of coated pits and plaques analyzed. The maximum recruitments of clathrin LCa-DsRed, cortactin-EGFP, Arp3-EGFP, or dynamin2-EGFP by plaques are statistically higher (*p*<0.0001) than recruitment by pits.

Cortactin appears at endocytic clathrin structures during late stages of coat formation in mammalian cells [8],[10]. We compared the recruitment of cortactin to clathrin plaques and pits, using Swiss 3T3 cells stably expressing LCa-DsRed and transiently expressing cortactin-EGFP. Cortactin recruitment to coated plaques was low, but it increased steadily during growth, with a peak just prior to the onset of uncoating (Figure 5). In contrast, coated pits had very low levels of cortactin fluorescence throughout the growth phase (Figure 5). The overall cortactin level in coated pits was approximately 4-fold lower than in plaques. Our results with coated plaques are similar to those reported by Merrifield and colleagues when analyzing relatively long-lived clathrin structures at the adherent surface of the cells [10]. Because cortactin activates Arp2/3, we also followed recruitment of Arp2/3 to endocytic clathrin structures in Swiss 3T3 cells stably expressing LCa-DsRed and Arp2/3-GFP. Arp2/3 was barely detectable at coated pits. In contrast, following low levels of recruitment during most of the lifetime of a coated plaque, Arp2/3 peaked at the onset of uncoating (Figure 5B). This observation is consistent with the acute recruitment of Arp2/3 seen at the time of invagination and uncoating of the long-lived clathrin structures analyzed by Merrifield and coworkers [37].

Based on these observations, we suggest that assembly of actin and its associated proteins is necessary for both early and late phases in the formation of a clathrin-coated plaque, but that it is not critical for the formation of clathrin-coated pits.

### Comparative Dynamics of Clathrin-Coated Pits and Plaques

We analyzed the incorporation of different coat constituents by complementary optical imaging methods. The fluorescent spots representing coated pits, imaged by spinning disk confocal microscopy, WF, or TIR fluorescence illumination, are of diffraction-limited dimensions, whereas the plaques give brighter spots that are often larger than the diffraction limit and that change shape during their extended lifetime (compare the time series of the representative pit and plaque in Figure 1C; see also Video S4). We analyzed temporal differences between the two classes of coated structure, by applying a power-spectrum analysis to detect principal frequency components in the time course of fluorescence variation. The data shown in Figure S2 were obtained by spinning disk confocal imaging recorded from the adherent surface of LCa-DsRed–expressing Swiss 3T3 cells. The primary difference between the two structures is that the fluctuations of fluorescence intensity in mature plaques are short-lived in comparison with the lifetime of coated pits. Dissociation of coats of pits [5],[14] or of plaques (this study; [10]) represents an endocytic event. Detailed analysis of the time-lapse series used to generate the data in Figure 2 shows 11 dissolutions of plaques and 62 dissolutions of pits occurring in 308 µm^2^ of bottom membrane during a period of 250 s (50 frames), indicating that the contribution of plaques to endocytosis is relatively small.

**Figure 6:**
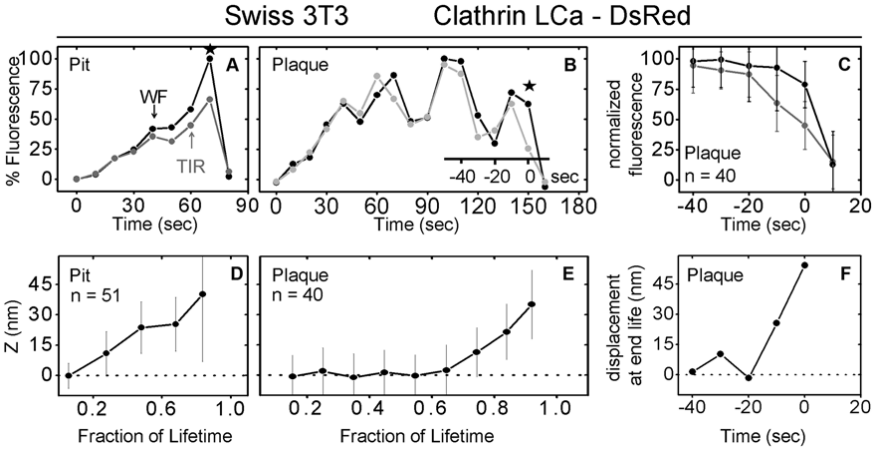
Movement of clathrin-coated pits and coated plaques into the cell interior. (A and B) Fluorescence intensity profile from a single canonical coated pit (A) or coated plaque (B) acquired from a Swiss 3T3 cells stably expressing clathrin LCa-DsRed by alternating TIR and WF illumination. The clathrin structures were imaged as they assembled on the bottom surface of the cell in direct contact with the coverslip. The TIR/WF cycles were acquired at 10-s intervals using exposures of 200 and 1,000 msec taken 200 ms apart. The WF fluorescence plot was normalized to its maximum fluorescence value to correct for variations in the intensity, whereas the TIR signals were normalized to that of the WF at the early stages of coat formation but allowed to diverge as the coat was formed. This corrects for differences in illumination and number of fluorophores in the spot [14]. Stars denote the endpoint used to calculate the lifetime. (C) Summary of TIR and WF data collected from all plaques during the last 40 s before their dissolution, with both datasets normalized to their corresponding maximum intensity, as in the work of Merrifield et al. [9]; each time point represents the average plus or minus the standard deviation (*n* = 40 plaques). (D and E) The average axial position (*z*, nm) for the ensemble of clathrin LCa-DsRed captured in the coat of pits (D) or plaques (E), plotted as a function of their respective lifetimes as calculated using, 
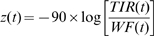
 with an evanescent TIR field penetration of 90 nm determined experimentally [14]. Each point represents the average plus or minus the standard deviation for all pits (*n* = 51) and plaques (*n* = 40). The coats of canonical pits shift continuously inward as they assemble, whereas plaques move abruptly inwards only just before dissolution. (F) The average axial position (displacement just before dissolution) of the clathrin plaques as calculated from average intensities presented in (C) using the same equation as in (E). This method of calculation is identical to the one used in Merrifield et al. [9]; using this method, we detect a similar movement away from the surface at the end of the plaque lifetime, as shown in (E).

We used a combination of WF and TIR illumination to examine the displacement of coated structures along the optical *z*-axis as a function of time (Figure 6). The signal acquired by WF fluorescence microscopy is proportional to the total number of fluorophores, whereas the TIR signal is also inversely related to the distance from the coverslip of the same fluorescing object. The TIR-to-WF ratio obtained by this approach (first used with clathrin structures by Merrifield and coworkers [9]) is proportional to the average distance from the coverslip of the clathrin assembly as it moves inward during endocytosis. We carried out the relevant measurements on images recorded from the bottom surface of Swiss 3T3 cells stably expressing LCa-DsRed (Figure 6). The clathrin centroid of a conventional coated pit moves steadily inwards into the cell and away from the coverslip (Figure 6A and 6D), as expected for a coat that grows continuously from a shallow dome into a larger, nearly spherical coated vesicle. Plaques also move inward (i.e., away from the coverslip), but only during the last 10–20 s of their lifetime (Figure 6B, 6C, 6E, and 6F). The plaque properties are precisely those outlined by Merrifield and colleagues in their description of endocytic clathrin structures in the adherent surface of the same Swiss 3T3 cell line [9],[10].

**Figure 7:**
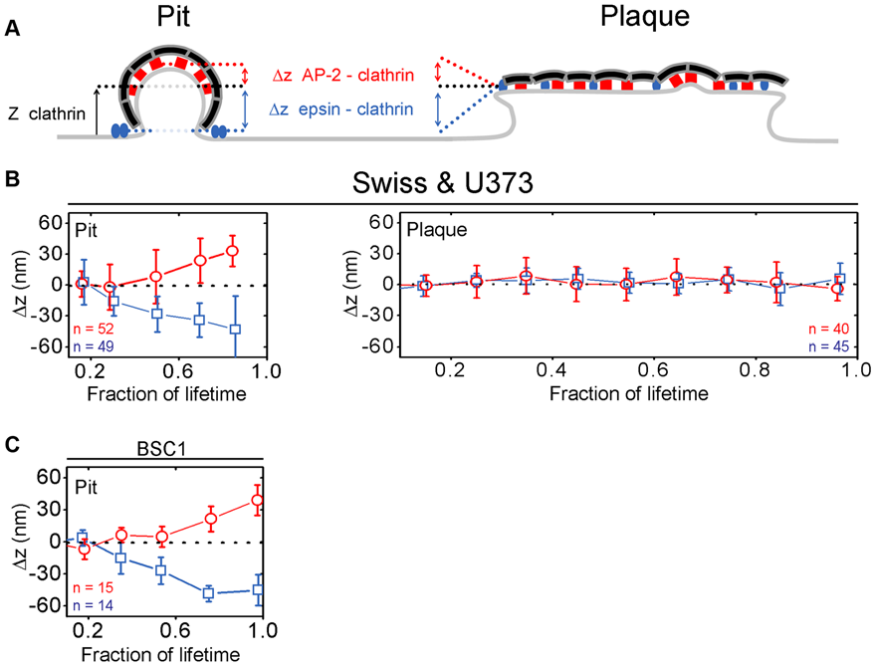
Relative positions of clathrin, AP-2, and epsin in coated pits and plaques. (A) Models representing the distribution of clathrin, AP-2, and epsin during the formation of a canonical clathrin-coated pit or a clathrin-coated plaque. (B) Difference in the axial positions of clathrin Tomato-LCa or clathrin LCa-DsRed with respect to AP-2 σ2-EGFP (Δ*z*, red) or to epsin1-EGFP (Δ*z*, blue) determined for individual pits or plaques selected for DiNa analysis in U373 astrocytes (52 pits and 40 plaques) or Swiss 3T3 fibroblasts (49 pits and 45 plaques) cells. The difference of axial position within each pit is plotted as a function of time; to facilitate the comparison between pits of different lifetimes, the time component is normalized as a fraction of each lifetime. DiNa results were obtained from data acquired three Swiss and three U373 astrocytes. The axial separations become statistically significant (*p*<0.0001) when the pits reach a maturity of 50% or more. (C) Difference in the axial position of clathrin Tomato-LCa with respect to AP-2 σ2-EGFP (Δ*z*, red) or to epsin1-EGFP (Δ*z*, blue) determined for 15 and 14 individual pits selected for DiNa analysis in BSC1 cells, respectively. Similar DiNa results were obtained from data acquired in three additional Swiss and three additional astrocytes (unpublished data). Error bars in (B) and (C) indicate plus or minus the standard deviation.

We have shown that in BSC1 cells, the AP-2 adaptor complex is recruited together with clathrin during the early stages of endocytic coated-pit formation (up to two-thirds of the total assembly time), but that adaptor incorporation then levels off during the final third of the process [14]. We devised an approach we have called *differential evanescence nanometry* (DiNa)—an extension of the TIR-to-WF ratio method described in the preceding paragraph—to measure the relative *z*-axis positions of two fluorophores with a precision of approximately 10 nm [14]. We determined the relative displacements of clathrin (tagged with Tomato-LCa) and AP-2 (tagged with σ2-EGFP) and showed that AP-2 is enriched in the “upper” hemispheres of the coated pits that form on the adherent surface of a BSC1 cell [14] (Figure 7C). Thus, the oldest part of the pit contains most of the adaptor, consistent with the decline in its incorporation rate, relative to that of clathrin, as the pit nears completion. These observations help explain the asymmetric distribution of density, in cryoelectron tomograms of brain-derived coated vesicles, which we attribute to adaptor molecules [19].

**Figure 8:**
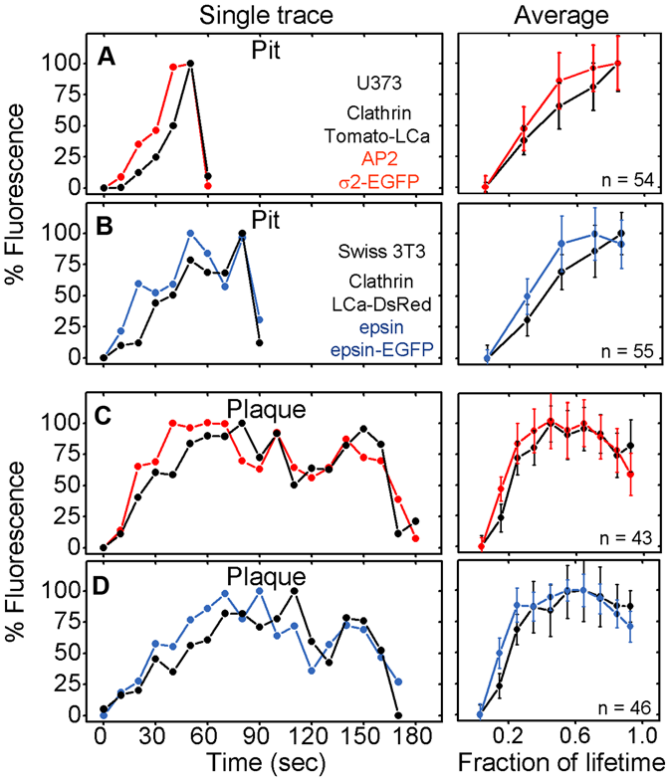
Recruitment of AP-2 and epsin into clathrin-coated pits and plaques. WF fluorescence intensity profiles obtained from time-series images of clathrin assemblies forming at the bottom surface of cells using 1-s simultaneous exposure with 10-s intervals. The data were obtained from (A and C) U373 astrocytes stably expressing AP-2 σ2-EGFP and transiently expressing Tomato-LCa, or (B and D) Swiss 3T3 fibroblasts stably expressing Clathrin LCa-DsRed and transiently expressing epsin1-EGFP. Left panels represent examples from single pits or plaques as a function of time. Right panels represent averages from the complete data sets normalized to the length of each lifetime. Each point represents average plus or minus the standard deviation; the data from the astrocytes presented in (A) and (C) were obtained from the three cells; the data from the fibroblasts presented in (B) and (D) are also from three cells; *n* is the number of analyzed pits and plaques.

We used U373 astrocytes to extend these observations to a cell type in which coated plaques could be studied. We confirmed that in these cells, as in BSC1 cells, AP-2 incorporates selectively during the earlier stages of conventional coated-pit formation (Figure 8A). DiNa measurements in U373 cells further showed that for late-stage coated pits, the *z*-centroids of the clathrin and AP-2 distributions are displaced (Figure 7B, pit), just as in BSC1 cells (Figure 7C, pit). Coated plaques do not show the same differential pattern of incorporation and displacement. Instead, clathrin and AP-2 co-incorporate at all stages (Figure 8C), and the *z*-centroids of clathrin and AP-2 remain coincident throughout the coated-plaque lifetime, even just prior to plaque dissolution, after the coat of the plaque has moved toward the cell interior (Figure 7B, plaque). Thus, clathrin and AP-2 move together into the cell interior as the plaque assembles and buds, carrying endocytic traffic toward endosomal compartments.

### Recruitment of Epsin and Dynamin

Epsin has a modular structure, including an N-terminal homology (ENTH) domain that binds phosphoinositides, ubiquitin interaction motifs that bind ubiquitin-containing proteins, and binding sites for clathrin, AP-2, and Eps15 [53],[54]. Like Eps15 [55], epsin does not incorporate into a budded coated vesicle [53], even though its accumulation parallels that of clathrin during coat formation [13],[16]. Epsin accumulates with clathrin during the formation of pits or plaques in Swiss 3T3 (Figure 8B and 8D) or BSC1 (unpublished data) cells transiently expressing Epsin1-EGFP and stably expressing either LCa-DsRed or Tomato-LCa. DiNa of conventional coated pits in Swiss 3T3 cells (Figure 7B, pit) or in BSC1 cells (Figure 7C) shows that as the pit matures, most epsin molecules remain at the growing edge of the coat, in association with the cell membrane, which in turn adheres to the coverslip. Localization of epsin at the edge of a coat is in accord with the enrichment of epsin and Eps15 at the growing edge of a coated pit, seen by immunoelectron microscopy.[54]–[56]. In a coated plaque, however, the average axial positions of clathrin and epsin always overlap, not only during the full lifetime of the plaque, but also during the final stages prior to dissolution, when the clathrin coat moves inward (Figures 6E and 7A and 7B, plaque). This observation agrees with the absence of a preferred location for epsin in extended arrays of clathrin observed by electron microscopy [54],[57].

Dynamin associates with coated pits during the growing phase of the coat and then in a stronger burst after completion and just prior to uncoating (Figure 5C) [22],[39]. Dynamin associates similarly with plaques, most prominently just before uncoating (Figure 5C), as previously described [9].

## Discussion

### Pits and Plaques

**Figure 9:**
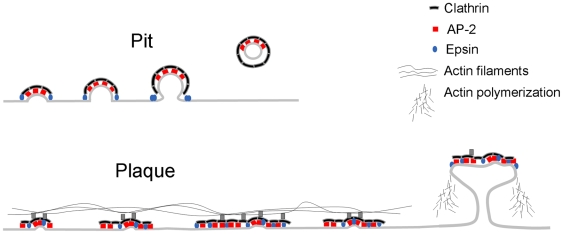
Models representing the sequential formation of a clathrin-coated pit and a coated plaque. Coated pits are small, rapidly forming structures that by progressive recruitment of clathrin, adaptors, and other regulatory proteins deform the underlying membrane as they bud inward to form coated vesicles. Coated plaques are larger, less dynamic, and relatively planar structures that move uniformly inward from the cell surface shortly before the membrane pinches off. Presence and/or remodeling of the actin cytoskeleton is essential for the formation, inward movement, and dissolution of clathrin-coated plaques, but it is not required for formation and budding of the rapidly growing clathrin-coated pits.

Our analysis of endocytic clathrin coats in living cells in culture shows two distinct kinds of coated structure (see model, Figure 9). One type, seen at both the free and adherent surfaces of the cell, corresponds to the canonical coated pits. Its coat assembles progressively into a curved lattice, which deforms the membrane as it grows. Pinching off of a coated vesicle (dynamin dependent but actin independent) and coat disassembly complete the clathrin cycle. The other type, seen only at the adherent surface, corresponds to clathrin-coated plaques. The coat is roughly planar (or moderately domed, as suggested by some of Heuser's micrographs [27],[29],[57]); after some minutes, it moves inward toward the cell interior, bringing with it a portion of the underlying membrane, which buds off in an actin- and dynamin-dependent process. In the presence of an actin depolymerizer, latrunculin A, new coated plaques do not form. Those already formed continue to accumulate, but they freeze in place and do not bud. Ablation of the clathrin-Hip1R interaction, by mutation or depletion of the light chains, or by depletion of Hip1R also prevents coated-plaque formation. Coated plaques tend to be larger and longer-lived than coated pits, and they show more variable assembly dynamics. As we discuss below, a key result is the recognition that pits and plaques have distinct functional properties. Understanding the differences helps reconcile the apparently disparate properties of clathrin-dependent endocytosis in various cell types and at various cellular surfaces.

Our imaging experiments used improved optics and other instrumentation that allowed longer-term imaging with minimal photobleaching. We recorded data from both the free and adherent surfaces of three different types of cells, and we excluded from the analysis all clathrin-containing clusters for which a high mobility indicated endosomal localization [5]. We also used AP-2 and epsin to restrict our analysis to coats at the plasma membrane. Our experiments show that clathrin plaques are present only at the adherent surfaces of Swiss 3T3, U373 astrocytes, and HeLa cells and that they are essentially absent from BSC1 cells. Canonical coated pits form with similar characteristics at both free and adherent surfaces of all four cell types. A subset of the coated pits form at preferred sites, or hot spots, but we find such sites primarily at the adherent surface of Swiss 3T3 cells and astrocytes, as described previously in COS cells [6].

Assigning previous observations to one or the other class of objects resolves apparent discrepancies in the published literature. Merrifield and coworkers [9],[10] combined TIR and WF fluorescence microscopy to examine formation of clathrin structures, tagged with LCa-DsRed, at the adherent surface of Swiss 3T3 cells. Most of their reported structures have a constant fluorescence signal and are relatively long-lived (>200 s). These and other published tracings [13],[15],[38],[43] show the final interval in the lifetime of these objects but do not illustrate the growth phase. A characteristic of all the structures they examine is an abrupt inward movement coincident with membrane budding shortly before dissolution of the coat. These steps represent bona fide endocytic events, by the criterion that entrapped transferrin-receptor, tagged with a pH-sensitive fluorophore, does not respond to pH changes in the medium once the structure has begun to uncoat [10]. We have now been able to use a similar TIR-WF protocol, but with substantially longer acquisition periods, to capture unambiguously the complete lifecycle of all clathrin structures, whether long or short. We specifically included in our analysis the Swiss 3T3 cell line studied by Merrifield et al. We confirmed that all the long-lived structures at the adherent surface indeed move inward abruptly just before losing their coats, in contrast to the continuous inward movement of clathrin and AP-2 during the growing phase of a canonical coated pit. We conclude that the long-lived endocytic clathrin structures analyzed by Merrifield et al., are the same as the clathrin-coated plaques described here. They are probably also equivalent to the persistent clathrin-containing structures observed by TIR in primary adipocytes [28]. These assemblies, which represent more than half the total clathrin signal at any time point, have variable size, are on average 2.5 times larger than pits, and have lifetimes longer than 3 min. Taking into consideration that plaques are relatively larger and that they invaginate at a considerably smaller frequency than pits, and including the contribution of pits forming on the free surface, we estimate that plaques carry approximately 11% of the transferrin uptake mediated by clathrin. This estimate is in agreement with the insignificant change of transferrin uptake in cells lacking plaques due to perturbations in the interaction between clathrin light chains and Hip1R (this study; see also [47]) or due to Hip1R ablation by RNAi [34].

Do the long-lived coated structures correspond to the gently domed, extended arrays of clathrin seen on the adherent surface of unroofed cells by freeze-etch electron microscopy? Several lines of evidence argue that they do. First, the extended arrays are seen only on the cytoplasmic surfaces of adherent plasma membranes containing plaques as detected by live-cell imaging—e.g., the HeLa cells imaged in Figure 1—and not on the corresponding surfaces of cells—e.g., the BSC1 cells in Figure 1—that do not contain plaques. Second, the sizes of the arrays seen by electron microscopy and the plaques described here are similar. The largest of the hexagonal arrays in published micrographs are about 500–1,000 nm in diameter, and most are smaller (this study and [24]–[27]). The plaques have dimensions that range from diffraction limited to several hundred nanometers. Third, the DiNa measurements described here show that the coated plaques we have characterized are essentially flat, as are those seen by electron microscopy. Fourth, extended fluorescent patches induced during InlB-dependent uptake of *Listeria monocytogenes* [58] probably correspond to hexagonal flat arrays seen by microscopy during engulfment of latex beads by macrophages [24].

Why do the available electron micrographs of thinly sectioned samples not show deeply invaginated plaques? A simple explanation is provided in the graphical representation presented in Figure S6; the data were obtained from a time-lapse series, and show, for every frame of the video, the number of structures scored as pit or plaque, and the number of pits scored as deeply invaginated (the last 10–15 s of their lifetime) or plaques scored as displaced from the substrate (by 100–150 nm during the last 5 s of their lifetime). This analysis reveals detection of a total of 799 structures scored as pits and 544 as plaques, with 197 images of deeply invaginated pits and only 11 substantially displaced plaques. Thus, the likelihood of visualizing deeply invaginating plaques by electron microscopy of thin sections is extremely low, and presently it is unrealistic to expect acquisition of a sufficient number of images to draw confident conclusions.

### Coated Plaque Assembly

Spontaneous clathrin assembly under suitable conditions in vitro yields closed structures that range in diameter from 60 to 200 nm. Reconstructions from cryoEM images of D6-barrel and tetrahedral coats show that curvature is built into the pucker at the apex of a triskelion and that side-by-side packing of the legs can vary so as to create much flatter lattices [21]. The hexagonal arrays seen by electron microscopy on the inward-facing surface of membranes apposed to plastic substrates or bacterial cell walls are quite imperfect, with frequent defects, as if their enforced planar assembly had introduced strain, just as expected if we were to extrapolate to zero curvature the variation in lateral contacts seen in more sharply curved coat structures. Thus, the extended flat arrays could arise because adhesion to the external substrate resists introduction of curvature, and essentially planar arrays grow until the accumulated strain is best compensated by a defect in the lattice. But the requirement of an intact light-chain-Hip1R-actin linkage for plaques to appear and the correlation of plaques with cell motility both suggest that cytoskeletal interactions may help direct coated plaque formation. AP-2 is also essential, for both coated plaques and canonical coated pits at the plasma membrane (this paper). It is therefore possible that pits and plaques initiate similarly, through an AP-2 dependent mechanism, but that the organization of the actin cytoskeleton dictated by adhesion to a substrate redirects clathrin assembly into planar arrays. To outline a specific mechanism for this redirection would require a more detailed picture than now available of how Hip1R associates with its partners. We note that initiation of clathrin coats that is AP-2 independent can also occur, as the *Listeria*-directed arrays neither incorporate AP-2 nor require it [59].

### Epsin

Epsin accumulates along with clathrin during assembly of a canonical coated pit. Our measurements of the relative *z*-displacement of epsin and clathrin show that the former remains in the plane of the surrounding membrane as the coated pit invaginates. That is, most of epsin does not move into the domed region, nor is it present in the mature coated vesicle. It therefore must accumulate largely around the rim of the pit, as does its partner, Eps15 [55],[56]. A limited number of published [54],[57] and unpublished electron micrographs (L. Traub, personal communication) suggest an accumulation of epsin on the rim of the pits, but the number of available images are not sufficient to draw a firm conclusion. At very high concentrations, epsin can tubulate liposomes, apparently by insertion of an N-terminal amphipathic helix into the outer leaflet of the bilayer [60]. This property has led to the suggestion that a function of epsin in coated-pit formation is to stabilize membrane curvature [60]. The location of most of the epsin, at the base of the invaginating pit, a position at which there is little net curvature, is not consistent with this proposal. Moreover, epsin is present in the essentially uncurved coated plaques, where its fluorescence intensity fluctuates in parallel with clathrin, and it moves inwards together with clathrin when the plaque internalizes. Because there is no curvature, and hence no differential *z*-displacement, we cannot tell from the DiNa measurements whether epsin is present throughout the plaque, or mostly at its rim as in a canonical coated pit. Published electron micrographs suggest that epsin may be distributed throughout the lattice [54],[57], but the studies have not been extensive enough to draw firm conclusions. Epsin contains multiple ubiquitin-interacting motifs (UIMs), and it is believed to facilitate incorporation of polyubiquitinated cargo into clathrin structures [57], whereas association of epsin with monoubiquitinated substrates is reported to exclude interaction with clathrin coats [61]. How epsin's putative function as a receptor for ubiquitinated cargo relates to its localization remains to be determined.

### Membrane Budding and Uncoating

The clearly defined, inward shift of a coated plaque, which invariably occurs shortly before dissolution, depends on actin and dynamin. As this shift is uniform across the plaque, there is no need to invoke any reorganization of the clathrin lattice into a curved structure [18],[29]; membrane uptake is clearly determined by other processes. Local reorganization of the actin cytoskeleton is the best candidate for the driving force of this invagination. There is considerable evidence that the cortactin-activated, Arp2/3 branching mechanism may have a role [10],[15],[34]. For example, Merrifield et al. [10] report cortactin recruitment at late stages of coat formation, and they link its arrival to internalization. Engqvist-Goldstein et al. [34] show that recruitment of Hip1R into long-lived clathrin coats (which we interpret as coated plaques) follows clathrin accumulation (as might be expected from its direct interaction with light chains), whereas cortactin peaks just before internalization. Our data on coated plaques are in full agreement with these observations. As Hip1R is essential for plaque initiation, we cannot determine whether its linkage to actin is also critical for internalization.

Our data suggest that uncoating of a plaque proceeds by an auxilin- and Hsc70-dependent mechanism, as for canonical coated vesicles. Auxilin is recruited during the last seconds of a coated plaque lifetime, but it is also incorporated in variable amounts during the course of assembly. It is possible that auxilin and Hsc70 catalyze exchange of clathrin into and out of the plaque during its lifetime [43].

### Plaques, Actin, and Yeast Cells

Ample genetic evidence points to a pathway that requires both clathrin and actin for endocytosis in yeast cells (reviewed in [30]). Moreover, live-cell fluorescence microscopy shows that clathrin, actin, and some of their interacting proteins are recruited to distinct sites at the plasma membrane, beginning with clathrin and Las17 (a regulator of Arp2/3) followed by actin and ending with a number of kinases and phosphatases, which facilitate efficient disassembly of different endocytic coat proteins [62]. This sequence of molecular associations parallels inward movement of the clathrin fluorescent spot [4],[11],[62]. Tubular invaginations of up to 50 nm in diameter and 180 nm in length, decorated with clathrin at their tips, can be seen emanating from the plasma membrane [63]. Thus, actin-based activities appear to drive a partially coated clathrin structure inward. Clathrin may provide the membrane anchor, and some additional activity may then drive membrane scission. We suggest that the endocytic pathway and its mechanisms characterized in yeast are closely related to the uptake of coated plaques in more complex eukaryotic cells.

### Functions of Clathrin-Coated Pits and Coated Plaques

The endocytic functions of canonical coated pits have been well documented. The canonical structures are the principal carriers for uptake of transferrin receptor and many other membrane-inserted proteins and for rapid reuptake of membrane components at synapses [2],[3]. Coated plaques can also take up transferrin (and presumably other receptor-bound ligands) [9],[10],[28], but their principal physiological role remains to be determined. When they do form, coated plaques appear to require contact with an external substrate. Conversely, the increased cell motility we see when coated plaques are ablated suggests that loss of plaques is linked to a loss of firm attachment. The close interplay between the actin and clathrin systems responsible for plaque internalization may also have been hijacked by invaders such as certain bacteria [59] and by large viruses [36]. Clathrin, dynamin, actin, and Arp2/3 are all required for invasion by *L. monocytogenes* and for pinocytosis of beads coated with the invasin InlB [58],[59],[64]. Extended arrays of clathrin have been seen on the adherent surface of osteoclasts [25],[26], on the cytoplasmic face of the invaginated cell membrane during bead uptake [24], and extended clathrin arrays are also present on endosomes [65]. The latter arrays are not associated with the usual heterotetrameric adaptor proteins.

In summary, we have established criteria for identifying coated plaques, and we have described many of their properties as detected by live-cell fluorescence microscopy. The properties of coated plaques, as worked out by quantitative analysis of our live-cell data, are distinct from the properties of canonical coated pits and are fully consistent with all published observations of clathrin-coated structures by electron microscopy, including the new electron microscopy data presented here. The participation of actin in coated-plaque internalization is the most obvious difference between these modes of membrane remodeling, but we have documented others, including the dynamics of coat assembly and the relationship to processes such as adhesion and motility. Clathrin is clearly a scaffold for a number of mechanistically distinct, membrane-associated process.

## Materials and Methods

### Preparation of Plasmids, Expressor Cells, Immunofluorescence, and RNAi

Swiss 3T3 cells expressing LCa-DsRed were kindly provided by Dr. W. Almers, BSC1, and U373 cells expressing σ2-EGFP were previously characterized in [5],[22]. LCa-DsRed was made by fusing rat LCa DNA to the 5′ end of the coding sequence for DsRed (Clonetech). Epsin-EGFP was created by fusing the rat epsin-1 DNA to the 5′ end of the coding sequence of EGFP (Clonetech). Tomato-LCa was created from rat LCa as described in [22]. BSC1 cells stably expressing epsin-EGFP were obtained by transfection using Fugene 6 (Roche Applied Science). A single clone of low-expressing Epsin-EGFP was maintained by selection with G418. All cells were maintained at 37°C and 5% CO_2_ in Dulbecco's modified Eagle medium (DMEM) with 10% fetal bovine serum and 0.4 mg/ml G418. As a control, BSC1 cells were transfected with LCa-DsRed and imaged 48 h after transfection. The dynamics of the clathrin spots under these conditions (Figure S3) show no difference to those in EGFP- or Tomato-LCa–expressing cells. U373 astrocyte cells stably expressing σ2-EGFP were used in conjunction with transient expression of Tomato-LCa and imaged after 48 h of transfection. Swiss 3T3 cells stably expressing LCa-DsRed were used in conjunction with transient expression of epsin-EGFP and imaged after 48 h of transfection. Cells transiently expressing cortactin-EGFP (gift of Dr. D. Drubin), EGFP-LCb, or EGFP-LCb-EED/QQN (gift of Dr. P. McPherson) were also imaged after 48 h of transfection. All transfections were carried out using Fugene 6 according to manufacturer instructions. For immunofluorescence, a monoclonal antibody (mAb), CON.1, specific for clathrin LCa and LCb was generated from hybridoma cells obtained from the American Type Collection. Prior to staining, the cells were fixed with 3.75% paraformaldehyde, and then sequentially labeled with CON.1 and a Cy3-labeled antimouse polyclonal antibody. Depletion of the clathrin light chains was achieved by sRNAi-mediated gene silencing experiments by transfecting U373 astrocytes stably expressing σ2-EGFP with a mixture containing 200 nM of LCa oligo (D-004002-01; Dharmacon) and 200 nM of LCb oligo (D-004003-03; Dharmacon) with Oligofectamine (Invitrogen); the treatment was repeated after 48 h, and the cells were replated after 62 h onto glass coverslips for imaging 10 h after. A scrambled oligo sequence (Dharmacon; gift from Dr. J. Liberman) was used as control. The efficiency of LCa and LCb depletion was determined by measuring the loss of immunofluorescence using CON.1 specific for both light chains. Depletion of Hip1R was achieved by sRNAi-mediated gene silencing experiments by transfecting U373 astrocytes stably expressing σ2-EGFP with 200 nM of a mixture containing Hip1R oligos (L-027079-00; Dharmacon) and with Oligofectamine (Invitrogen) or by first transfecting U3T3 astrocytes with the mixture of Hip1R oligos, which where then transfected 2 d later with EGFP-LCb.

### Handling of Cells Prior to Imaging

Roughly 20,000–40,000 cells were plated on glass #1.5 coverslips (25 mm in diameter) 10 h before the actual imaging experiment. Up to this point, the cells were maintained at 37°C and 5% CO_2_ in DMEM with 10% fetal bovine serum. Cells were imaged in Gibco's CO_2_ independent medium and in the absence of phenol red (Invitrogen). The temperature of the sample holder (20/20 Technologies) was kept at 37°C using a Peltier-controlled holding device. The holding device and the stage were surrounded by a custom-designed air-controlled environmental chamber kept at 33–35°C.

### Imaging

All spinning disk confocal imaging experiments were conducted by using the microscope setup previously described [5]. We further modified the microscope by insertion of a computer-controlled Spherical Aberration Correction unit (SAC; Intelligent Imaging Innovations), which drastically reduced the spherical aberration and thus increased the sensitivity. In addition, this setup was modified with lasers equipped at 473 nm and 561 nm (Cobolt) and 660 nm (Crystal Lasers). The lasers input to the fiber optic is controlled through a PCAOM AOTF module (Neos). The emission is collected after the spinning disk unit and passes through a dual view unit (Roper Scientific), equipped with a 565DCXR dichroic mirror and the HQ525/40 and HQ620/50 (Chroma) to separate the fluorescence of EGFP and Tomato/DsRed on two sides of the same CCD chip. The camera has been updated to a Cascade 512B (Roper Scientific), which is operated with multiplication gain and no binning. The Dual view unit and the AOTF allow for simultaneous or for fast switching between excitation with 473 and 561 nm. The sample is only exposed to the laser light while the CCD is acquiring, and is immediately turned off during the frame transfer time of the CCD, which can take 50–200 ms, depending on mode of readout. This shutoff is achieved by synchronizing the AOTF with the camera frame readout TTL signal (Intelligent Imaging Innovations). Using this setup, we have been able to image an exposure as low as 50–100 ms, reducing phototoxicity and allowing longer measurement times. Images were captured with SlideBook 4 software (Intelligent Imaging Innovations). All TIR/WF measurements were carried out using the conditions previously described [14].

### Image Processing

Clathrin clusters were identified using a sequence of deconvolution, 2D Gaussian and Laplacian filtering followed by thresholding, which created a mask as previously explained in [5],[22]. The masks were tracked using SlideBook 4 (Intelligent Imaging Innovations). The coordinates of the center of these masks versus time were exported along with the images. A MATLAB routine [14] was used to read the intensity profile for each object from the corresponding images. DiNa measurements were carried as previously described [14]. Clathrin-coated structures were selected according to the following criteria [5],[14],[22]: (1) the fluorescent objects appeared and disappeared within the time series; (2) the objects displayed the limited movement expected for membrane-bound clathrin structures in the horizontal plane during their growth phase (500 nm/lifetime); and (3) the objects did not collide with each other. Cell motility as a function of time was determined in cells imaged in WF bright illumination, by tracking the temporal change in the position of the center of the nucleus.

### Electron Microscopy

BSC1 and HeLa cells were plated on glass coverslips and grown for 4 h (BSC1) or overnight (HeLa) at 37°C and 5% CO_2_ in DMEM with 10% fetal bovine serum. To visualize the adherent surface of the cells, the samples were first broken open (“unroofed”) by standard methods, followed by chemical fixation, rapid freezing, deep etching, and rotary shadowing. [40]. The samples were visualized using a JEOL JEM 1200EX microscope operating at a nominal magnification of 30,000 and 80 kV, and imaged digitally with an AMTKK CCD camera.

## Supporting Information

Figure S1 **Effect of latrunculin A on the lifetime and dynamics of pits and plaques.** The kymograph is a schematic representation as a function of time of the effect of actin depolymerization on the lifetime of pits (gray) and plaques (black) in the adhered surface of a Swiss 3T3 cell stably expressing LCa-DsRed. The data derived from the time series obtained from a cell imaged using spinning disk confocal microscopy at 10-s intervals and 100-ms exposure time (Video S3). Acquisition of the time series was started at *t* = 0, and 5 µM latrunculin A was added at *t* = 540 sec. As shown before with similarly treated BSC1 cells [33], actin depolymerization results in the accumulation of clathrin structures on the plasma membrane but does not affect the dynamics of coated pits. Formation of new plaques ceases, and existing plaques completely freeze upon latrunculin A treatment.
(2.12 MB TIF)

Figure S2 **Intensity profiles of coated pits and plaques.** Concatenated tracings of different coated pits (gray) and plaques (black). The characteristic times for intensity fluctuations within the lifetime of a plaque are much shorter than the lifetime of a coated pit. A formal autocorrelation analysis (unpublished data) confirms this conclusion. Thus, the properties of a plaque cannot be attributed to those of a series of coated pits forming one after another at the same location.
(2.43 MB TIF)

Figure S3 **Formation of clathrin structures in BSC1 cells expressing clathrin LCa-DsRed.** Images from a time series acquired by spinning disk confocal microscopy from the adherent surface of a BSC1 cell transiently expressing clathrin LCa-DsRed. The cell was imaged 48 h after transfection, using 3-s intervals for a duration of 300-s and 150-ms exposures. The average lifetime of the clathrin structures (53±17 s) is similar to the average lifetime of canonical pits previously characterized in BSC1 cells stably or transiently expressing EGFP-LCa [5].
(1.68 MB TIF)

Figure S4 **Sizes and lifetimes of clathrin-coated structures forming at the cell surfaces.** In these double logarithmic plots, each dot corresponds to the duration (lifetime) and the corresponding size (maximum fluorescence intensity prior to dissolution) of a clathrin or AP-2 fluorescent spot recorded at the top or bottom plasma membrane of adherent cells. Each panel contains data obtained from three cells and includes the observations obtained from the single cells depicted in the experiments associated with Figures 2, 3, and 4. (A) Average lifetime of the canonical coated pits on the top of Swiss 3T3 cells stably expressing Clathrin LCa-DsRed is 54.6±29 s (*n* = 193). The maximum fluorescence intensity is 1,988±1,387. (B) Average lifetime of the canonical coated pits on the top of U373 astrocytes stably expressing AP-2 σ2-EGFP is 61±31 s (*n* = 130). The maximum fluorescence intensity is 1,220±607. (C) Average lifetime of the canonical coated pits on the bottom of BSC1 cells stably expressing AP-2 σ2-EGFP is 48±20 s (*n* = 620). The maximum fluorescence intensity is 1,232±483. (D) Average lifetime of the canonical coated pits and coated plaques on the bottom of Swiss 3T3 cells stably expressing Clathrin LCa-DsRed are 57±26 s (*n* = 200) and 269±130 s (*n* = 104), respectively. The corresponding maximum fluorescence intensities are 995±711 and 3,269±2,725. The differences in average lifetime or in maximum fluorescence intensity between pits and plaques in Swiss 3T3 cells are statistically significant (*p*<0.0001). (E) Average lifetime of the canonical coated pits and coated plaques on the bottom of U373 astrocytes stably expressing AP-2 σ2-EGFP are 62±22 s (*n* = 525) and 228±104 s (*n* = 110), respectively. The corresponding maximum fluorescence intensity are 1,447±769 and 2,503±1,270. The differences in average lifetime or in maximum fluorescence intensity between pits and plaques in astrocytes are statistically significant (*p*<0.0001). (F) Effect of latrunculin A on the lifetime of canonical coated pits and coated plaques on the bottom of Swiss 3T3 cells stably expressing Clathrin LCa-DsRed. The average lifetime and maximum fluorescence intensity of the canonical coated pits after treatment with 5 µM of latrunculin A are 92±40 s (*n* = 80) and 1,394±1,017, respectively. In contrast, plaques become stable, and their lifetimes are longer than 2,000 s (the extent of the time series). (G) Average lifetime of the canonical coated pits on the bottom of Swiss 3T3 cells stably expressing Clathrin LCa-DsRed and transiently over expressing EGFP-LCb-EED/QQN is 60±22 s (*n* = 204). The maximum fluorescence intensity is 2,242±1,344. Note the complete absence of coated plaques. (H) Average lifetime of the canonical coated pits on the bottom of U373 astrocytes transiently over expressing EGFP-LCb-EED/QQN is 92.3±33.1 s (*n* = 402). The maximum fluorescence intensity is 1,219±619. Note the absence of coated plaques. (I) Average lifetime of the canonical coated pits on the bottom of U373 astrocytes depleted of both clathrin light chains by sRNAi treatment is 60±25 s (*n* = 555). The maximum fluorescence intensity is 674±385. Note the complete absence of coated plaques.
(0.21 MB TIF)

Figure S5 **Relative contribution of plaques to the internalization of transferrin in U373 cells.** Cells transiently expressing EGFP-LCb or EGFP-LCb-EED/QQN for 3 d were incubated for 2 min at 37°C with 50 µg/ml Alexa 568 transferrin (Invitrogen). The cells were then washed with fresh DMEM containing 10% FBS for 5 min at 37°C and then fixed with 3% PFA. The whole volume of the cell was imaged using the spinning disk confocal microscope with 300-nm steps between consecutive imaging planes. The amount of internalized transferrin was estimated from the total fluorescence intensity; the small contribution of transferrin remaining at the cell surface was corrected by masking the outer border of the cell. Data shown for each condition are the average plus or minus the standard deviation, from five cells; the differences are not statistically significant.
(4.05 MB TIF)

Figure S6 **Scoring of events corresponding to pits and plaques at early and late stages of invagination or displacement.** The data illustrate the distribution of structures scored as pits or plaques in each of the imaging frames. Note that the actual number of pits and plaques traced during the duration of the time series is smaller. They also show the events scored as deeply invaginated pits, imaged during the last 10–15 s of their lifetimes, and of “lifted” plaques (displaced from the substrate), imaged during the last 5 s of their lifetimes. These data were obtained from the time series used to generate Figure 2. The video spans an interval of 250 s (50 frames) and covers 308 µm^2^ of adherent surface. A total of 799 structures were scored as pits and 544 as plaques; and they include 167 deeply invaginated pits and 11 lifted plaques.
(4.60 MB TIF)

Video S1 **Formation of clathrin-coated structures on the free surface of Swiss 3T3 cells.** Time-series images of clathrin assemblies at the plasma membrane on the top of a Swiss 3T3 fibroblast stably expressing clathrin LCa-DsRed imaged using spinning disk confocal microscopy at 5-s intervals and 100-ms exposure time. The video shows 68 frames (340 s). Notice that the mobility of the clathrin assemblies is in part due to overall movement of the cell; some structures are clathrin coats on mobile endosomes.
(0.62 MB MOV)

Video S2 **Formation of clathrin-coated structures on the adherent surface of Swiss 3T3 cells.** Time-series images of clathrin assemblies at the plasma membrane on the bottom surface in contact with the coverslip of a Swiss 3T3 fibroblast stably expressing clathrin LCa-DsRed imaged using spinning disk confocal microscopy at 5-s intervals and 100-ms exposure time. Some structures are clathrin coats on mobile endosomes.
(2.38 MB MOV)

Video S3 **Formation of clathrin-coated structures on the adherent surface of Swiss 3T3 cells.** Zoom region of Video S2 showing 68 frames (340 s). Examples of one hot spot containing three pits (#s 1, 2, and 3), one plaque (#4) and two coated pits (#5 and 6) are highlighted. Note that coated pit #6 is an object that is independent from coated plaque #4: although their points spread functions partially overlap for a brief moment, the corresponding centers always remain separated.
(2.64 MB MOV)

Video S4 **Comparison of coat dynamics between clathrin pits and plaques.** Time-series images of clathrin assemblies at the plasma membrane on the adherent bottom of a U373 astrocyte (left) and a BSC1 cell (right) stably expressing σ2-EGFP imaged by spinning disk confocal microscopy at 10-s intervals and 70-ms exposure time. The video shows 80 frames (800 s). U3T3 cells form pits and plaques whereas plaques are not observed in BSC1 cells.
(6.33 MB MOV)

Video S5 **Clathrin-coat dynamics at the adherent surface of HeLa cells depleted of AP-2.** Time-series images of clathrin in HeLa cells whose AP-2s were depleted by using sRNAi specific for μ2. The images were acquired by alternating two channels in order to register the fluorescent signals for the stably expressed σ2-EGFP (left panel) and the transiently expressed Tomato-LCa (right panel). The video was acquired by spinning disk confocal microscopy at 10-s intervals and 200- and 100-ms exposure times, respectively. The video shows 50 frames (500 s). The mobile clathrin-coated structures in the cytosol and perinuclear regions have the usual characteristics of endosome- or TGN-associated coats containing AP-1, AP-3, GGAs, or Hrs.
(4.09 MB MOV)

Video S6 **Effect of latrunculin A on the lifetime and dynamics of pits and plaques.** Time-series obtained from a Swiss 3T3 fibroblast stably expressing clathrin LCa-DsRed imaged using spinning disk confocal microscopy at 10-s intervals and 100-ms exposure time. Acquisition of the time series was started at *t* = 0, and 5 µM latrunculin A was added at *t* = 540 s.
(7.55 MB MOV)

## Abbreviations

DiNa: differential evanescence nanometry
LCa: light chain a
LCb: light chain b
RNAiRNA interference
sRNAi: short RNA interference
TIR: total internal reflection
WF: wide-field
